# Comparative Analysis of Conventional Ideal Button Versus New Ideal Button ZERO for Percutaneous Endoscopic Gastrostomy Catheter Replacement

**DOI:** 10.1002/deo2.70261

**Published:** 2025-12-10

**Authors:** Kazuya Miyaguchi, Hisashi Matsumoto, Yuki Shiko, Yoshikazu Tsuzuki, Maiko Osawa, Rie Shiomi, Keiji Yamamoto, Yohei Kawasaki, Hiroyuki Imaeda

**Affiliations:** ^1^ Department of Gastroenterology Saitama Medical University Moroyama Japan; ^2^ Department of General Internal Medicine Saitama Medical University Moroyama Japan; ^3^ Department of Biostatistics Graduate School of Medicine Saitama Medical University Moroyama Japan

**Keywords:** catheterization, endoscopy, gastrostomy, percutaneous endoscopic gastrostomy, postoperative complications

## Abstract

**Objectives:**

Regular exchange of percutaneous endoscopic gastrostomy (PEG) catheters is crucial for preventing infection and maintaining function; however, procedure‐related complications and patient discomfort remain major concerns. This study aimed to compare the clinical outcomes of the conventional Ideal Button with those of the newly developed Ideal Button ZERO (Olympus Corporation).

**Methods:**

In this retrospective observational study, we included 82 PEG catheter exchange procedures, performed in 42 patients. Because some patients underwent repeated exchanges, analyses were conducted on a per‐procedure basis. Patients were categorized into two groups: Group N (conventional to conventional Ideal Button exchange, *n* = 33) and Group Z (exchange to Ideal Button ZERO, *n* = 49). Group Z was further subdivided into subgroups Z1 (conventional to ZERO, *n* = 29) and Z2 (ZERO to ZERO, *n* = 20). The outcomes included the procedure time, complication rates (procedure‐related and postoperative), and family satisfaction score.

**Results:**

Procedure‐related complications occurred only in Group Z (0/33 vs. 6/49; 0% vs. 12.2%) (*p* = 0.076). Postoperative complication rates were similar between the groups (Group N 6/33 [18.2%] vs. Group Z 9/49 [18.4%], *p* = 1.00). However, procedure time was shorter in Group Z than in Group N (8.24 ± 5.21 vs. 6.14 ± 4.28 min, *p* = 0.049). Family satisfaction scores showed no significant differences between the groups (Group N: 3.88 ± 1.52 vs. Group Z: 3.94 ± 1.39, *p* = 0.854).

**Conclusions:**

The new Ideal Button ZERO showed a reduced procedure time; however, it revealed a trend toward higher procedure‐related complications without clear superiority over conventional devices. Improved proficiency with the new device may reduce complication rates, warranting further investigation as its adoption increases.

**Trial Registration:**

2025‐047

## 1 Introduction

Percutaneous endoscopic gastrostomy (PEG) is a standard therapeutic intervention for patients requiring long‐term enteral nutritional management [1]. With the progression of a super‐aging society, the prevalence of dysphagia continues to rise, leading to expanding indications for PEG procedures [2].

Regular PEG catheter exchange is recommended for preventing infection and maintaining function, typically performed at 3–6‐month intervals. However, the necessity of routine exchange remains debatable, with recent large‐scale studies reporting no significant differences in complication rates among cases with prolonged placements exceeding 6 months [3].

Internal bumper‐type PEG tubes exhibit high durability and do not require routine exchange. Up to 70% may remain in place for over 2 years. The European Society of Gastrointestinal Endoscopy does not recommend routine exchange; instead, it advocates individualized exchange intervals based on catheter material and patient condition [4]. Nevertheless, potential functional deterioration due to catheter degradation and the increased infection risk make appropriate exchange intervals an important clinical consideration.

PEG exchange procedures are associated with pain and discomfort during catheter removal and insertion, as well as with procedure‐related complications. Reported procedure‐related complications include bleeding, perforation, and infection, in addition to postoperative complications, such as stoma site infections and catheter dislodgement [5].

Anderloni et al. reported on PEG replacement performed for 356 patients; the 30‐day mortality rate was 1.8%, while complications occurred in 1.7% of the patients [6].

PEG exchange procedures are usually considered “simple procedures”; however, severe complications, such as gastrocolocutaneous fistulas, have been reported [7]. In a multicenter collaborative study in Japan, the incidence of intra‐abdominal misplacement during exchange was four of 961 cases (0.42%) [8]. Notably, tube dislodgement is a relatively common PEG‐related complication, with reported incidence rates ranging from 13% to 29%. Button‐type tubes have been proposed as one method to prevent tube dislodgement [9].

Technological innovations in medical devices have significantly contributed to improved patient safety and reduced healthcare workers’ burden. Various improvements have been implemented in the PEG field, including material enhancement, design optimization, and procedural simplification. However, the introduction of new medical devices involves a learning curve, potentially resulting in higher initial complication rates compared with those observed with conventional procedures [10].

**FIGURE 1:**
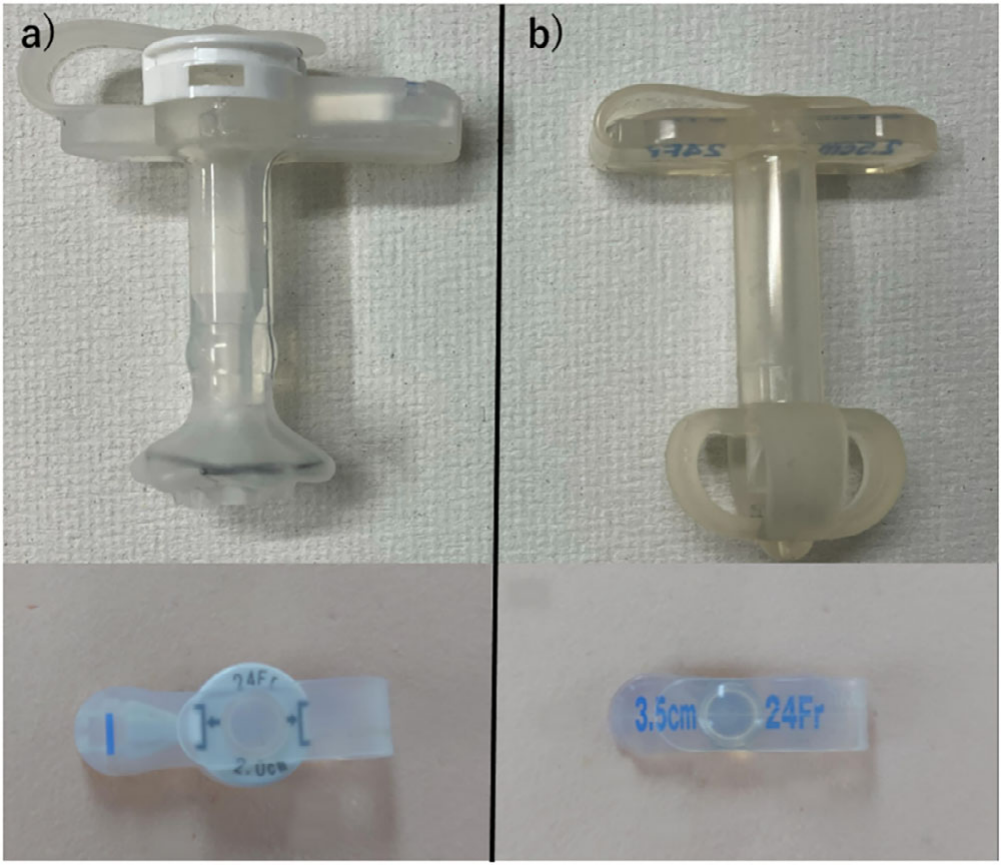
(a) IDEAL button ZERO device. (b) IDEAL button device.

To address these challenges, Olympus Corporation (Tokyo, Japan) launched the new Ideal Button ZERO in June 2023 (Figure 1). This device adopts an innovative mechanism that encapsulates the catheter tip within a capsule and has the following features:

1. Reduced fistula tract resistance during catheter removal through wire removal and bumper compression
2. Capsule‐based tip protection to reduce tract injury during catheter insertion
3. Simplified insertion technique with elimination of complex prior manipulations
4. Reduced patient burden through minimization of pain and discomfort during removal and insertion.

Despite these design advantages, comparative clinical studies between the new Ideal Button ZERO and the conventional Ideal Button are lacking, necessitating scientific validation of its safety and efficacy in real‐world clinical practice. Clinical evaluation of new medical devices requires a comprehensive assessment of complication rates, procedural efficiency, and patient satisfaction in clinical settings, beyond mere product specification improvements.

In this study, we compared complication rates, procedure times, and family satisfaction scores between the conventional Ideal Button and the new Ideal Button ZERO for PEG catheter exchange to clarify the clinical significance of the new device. The reason procedure time was chosen as the major outcome is that procedure time was used as an indicator of procedural efficiency, as reduced manipulation time may decrease patient burden and improve workflow efficiency.

## 2 Methods

### 2.1 Study Design

This single‐center retrospective observational study was conducted at Saitama Medical University Hospital. The study was approved by the institutional ethics committee (2025‐047).

### 2.2 Study Population

Forty‐two patients (82 procedures) who underwent PEG catheter exchange at the Department of Gastroenterology, Saitama Medical University Hospital, between April 1, 2020, and June 30, 2025, were included. Several patients underwent multiple exchanges during the study period; therefore, each exchange was analyzed as an independent procedure.

### 2.3 Inclusion and Exclusion Criteria

The inclusion criteria were as follows: age ≥18 years (any sex), requirement of routine PEG catheter exchange following initial PEG placement, previous exchange using either the Ideal Button or Ideal Button ZERO during the study period, and complete medical records containing the necessary data.

The exclusion criteria were as follows: PEG placement, emergency catheter exchange, and the presence of dementia or psychiatric conditions that prevented accurate pain assessment.

### 2.4 Group Classification

Patients were divided into two groups based on device exchange patterns (42 patients, 82 procedures).

- **Group N** (conventional Ideal Button to conventional Ideal Button): 33 cases
- **Group Z** (conventional exchange to Ideal Button ZERO): 49 cases
- Z1 (conventional Ideal Button to new Ideal Button ZERO): 29 cases
- Z2 (new Ideal Button ZERO to new Ideal Button ZERO): 20 cases

Group N included procedures using the conventional Ideal Button, whereas Group Z included exchanges where the Ideal Button ZERO was inserted, either after a conventional or another ZERO device. Since the launch of ZERO in 2023, we have been switching to ZERO except when patients or their families prefer otherwise.

### 2.5 Outcome Measures

Study endpoints included procedure time, complication rates (procedure‐related and postoperative), and family satisfaction score. Procedure time was used as an indicator of procedural efficiency, as reduced manipulation time may decrease patient burden and improve workflow efficiency.

Patient characteristics of interest were: age, sex, height, weight, performance status, underlying diseases, anticoagulant or antiplatelet use, time since PEG placement, and number of exchanges. Operator experience was recorded.

### 2.6 PEG Tube Characteristics

All PEG tubes were standardized to 24‐Fr in diameter and 3.0–3.5 cm in length, with no significant differences between groups.

### 2.7 Family Satisfaction

Family satisfaction with the user experience was assessed after 2 weeks using a structured 5‐point Likert scale (1 = very dissatisfied to 5 = very satisfied) by the attending nurse. The questionnaire evaluated procedural comfort and overall satisfaction.

### 2.8 Exchange Methods

PEG catheter exchange was performed using either a standard upper endoscope or a dedicated PEG scope, chosen at the endoscopist's discretion based on fistula maturity and visualization requirements. First, the scope was inserted into the stomach to confirm the already‐placed PEG tube, after which the PEG tube was replaced while monitoring the endoscopic screen.

### 2.9 Complication Definitions

Complication severity was assessed according to the Common Terminology Criteria for Adverse Events v5.0. Procedure‐related complications included bleeding, perforation, infection, catheter damage, and wire breakage. Postoperative complications included stoma site infections, subcutaneous migration, intraperitoneal migration, and catheter dislodgement.

### 2.10 Statistical Analysis

Continuous variables are expressed as means ± standard deviations, while categorical variables are presented as numbers (percentages). Between‐group comparisons of patient demographics were performed using Student's t‐test for continuous variables and Pearson's χ^2^ test for categorical variables. For primary and secondary endpoints, between‐group comparisons were performed using Student's t‐test for continuous variables and Fisher's exact test for categorical variables. Group differences with 95% confidence intervals were calculated for both continuous and categorical outcomes. A two‐sided *p*‐value of <0.05 was considered statistically significant. When patients underwent multiple PEG exchanges during the study period, each exchange was analyzed as an independent event. Given the small number of repeated cases (median 1 [range 1–3] per patient), intra‐patient correlation was not considered in this study. All analyses were performed using R version 4.5.0 (R Foundation for Statistical Computing, Vienna, Austria).

## 3 Results

### 3.1 Patient Demographics

During the study period, 82 PEG catheter exchanges (42 patients) were performed: 33 in Group N and 49 in Group Z (Z1: 29 cases; Z2: 20 cases).

The patient demographics characteristics are summarized in Table 1. The mean age did not differ significantly between the groups (Group N: 73.2 ± 17.4 years; Group Z: 65.2 ± 23.0 years, *p* = 0.16). No significant differences were observed in sex, height, weight, performance status, or underlying disease distribution between groups.

**TABLE 1:** Patient characteristics.

Variable	Level	*N* (*n* = 18)	Z (*n* = 24)	*p*‐value
Age (mean [SD])		73.2 (17.4)	65.2 (23.0)	0.16
Sex (%)				
	Male	8 (44.4)	12 (50.0)	0.70
	Female	10 (55.6)	12 (50.0)	
Height (mean [SD])		156.1 (9.7)	154.1 (10.4)	0.52
Body weight (kg) (mean [SD])		45.2 (10.7)	42.0 (9.8)	0.29
Performance Status 0–4 (mean [SD])		3.72 (0.46)	3.50 (0.78)	0.28
Underlying disease (%)				0.94
ALS		1 (5.6)	4 (16.7)	
Cerebrovascular disease		4 (22.2)	5 (20.8)	
Intellectual disability/developmental delay		1 (5.6)	3 (12.5)	
Parkinson's disease		4 (22.2)	5 (20.8)	
Dementia (FTD/Alzheimer's/others)		4 (22.2)	6 (25.0)	
Neuromuscular/myopathic disorders		2 (11.1)	3 (12.5)	
Others		2 (11.1)	3 (12.5)	
Antithrombotic medications (%)				0.07
None		14 (77.8)	23 (95.8)	
Aspirin		3 (16.7)	4 (8.2)	
Clopidogrel		1 (3.0)	2 (4.1)	

Abbreviations: ALS, amyotrophic lateral sclerosis; FTD, frontotemporal dementia; kg, kilogram; SD, standard deviation.

Anticoagulant/antiplatelet did not differ significantly between the groups (*p* = 0.07). Similarly, time since PEG placement, number of exchanges, and operator experience did not differ significantly between the groups (Table 2).

**TABLE 2:** Pre‐procedure baseline.

Variable	Overall (*n* = 82)	N (*n* = 33)	Z (*n* = 49)	*p*‐value
Length (mean [SD])	3.23 (0.57)	3.29 (0.57)	3.18 (0.57)	0.418
Period since PEG placement (mean [SD])	3.41 (3.53)	3.02 (3.53)	3.68 (3.55)	0.404
Number of exchanges (mean [SD])	6.87 (7.10)	6.09 (7.10)	7.39 (7.13)	0.421
Number of years of experience as endoscopists (mean [SD])	16.21 (9.40)	17.00 (9.82)	15.67 (9.17)	0.534

Abbreviations: PEG, percutaneous endoscopic gastrostomy; SD, standard deviation.

### 3.2 Procedure Time

The mean procedure time was significantly shorter in Group Z than in Group N (8.24 ± 5.21 min vs. 6.14 ± 4.29 min; mean difference [Group Z − Group N]: −2.10 min, 95% confidence interval [CI]: −4.19 to −0.01, *p* = 0.049) (Table 3).

**TABLE 3:** Procedure comparison.

Variable	Overall (*n* = 82)	*N* (*n* = 33)	Z (*n* = 49)	*p*‐value
Procedure time (mean [SD])	6.99 (4.76)	8.24 (5.21)	6.14 (4.28)	0.049
Family staff satisfaction rating: 1–5 (1 = very dissatisfied to 5 = very satisfied) mean [SD])	3.91 (1.43)	3.88 (1.52)	3.94 (1.39)	0.854
Request to switch to a different type of PEG (%)				
Change requested	11 (13.4)	4 (12.1)	7 (14.3)	0.637
No change requested	71 (86.6)	29 (88.9)	37 (85.7)	
How to exchange 1EGD 2 PEGSCOPE (%)				
1	68 (82.9)	26 (78.8)	42 (85.7)	0.604
2	14 (17.1)	7 (21.2)	7 (14.3)	

Abbreviations: EGD, esophagogastroduodenoscopy; PEG, percutaneous endoscopic gastrostomy; SD, standard deviation.

### 3.3 Complication Rates

#### 3.3.1 Procedure‐related Complications

Procedure‐related complications occurred in 0% (0/33) of Group N and 12.2% (6/49) of Group Z, representing a non‐significant trend toward higher rates in Group Z (risk difference [Group Z − Group N]: 12.2%, 95% CI: −1.31–24.77, *p* = 0.076).

**FIGURE 2:**
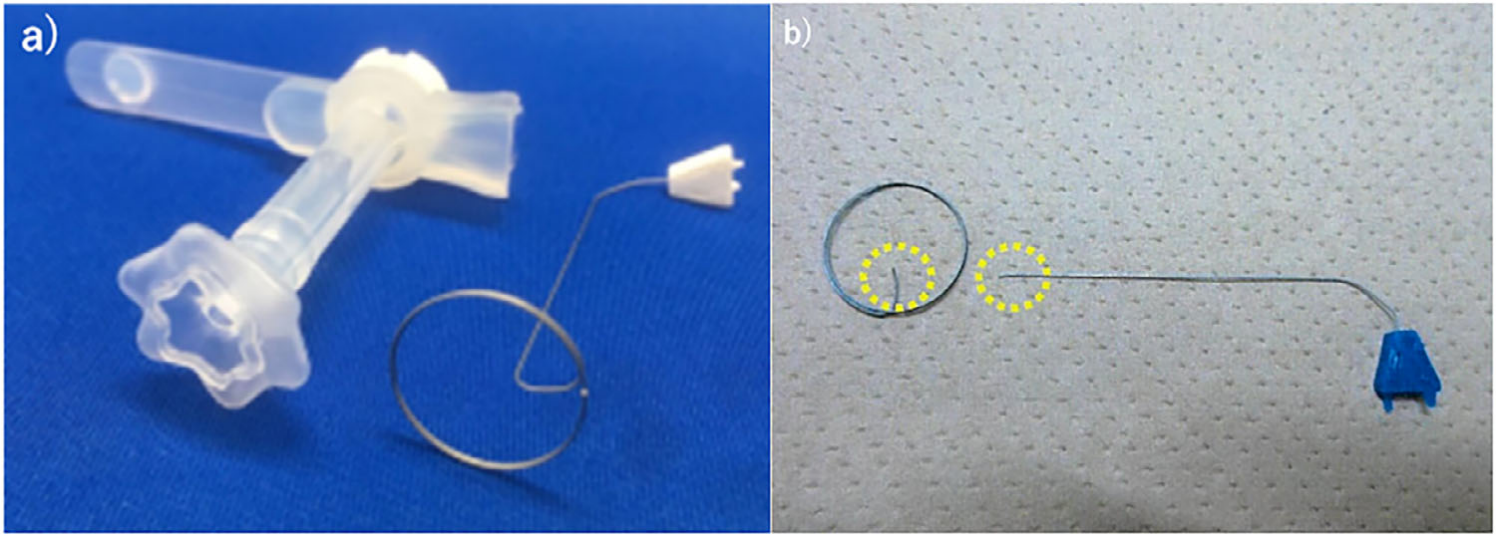
(a) Wire and button. (b) Examples of wire fracture (enclosed by a yellow dotted line).

Specific procedure‐related complications included wire breakage (Figure 2), bleeding, and inadequate bumper deployment, confirmed endoscopically in four (4.9%) cases, and one (1.2%) and one (1.2%) case, respectively. The single bleeding case was classified as “minor” and controlled endoscopically without transfusion or hospitalization. Both postoperative infections were mild cellulitis treated with oral antibiotics without hospitalization. All complications occurred in Group Z, with none reported in Group N (Table 4).

**TABLE 4:** Summary of complication rates.

Variable	Overall (*n* = 82)	N (*n* = 33)	Z (*n* = 49)	*p*‐value
*N*	82	33	49	
Occurrence of technique complications (%)				
Absent	76 (92.7)	33 (100.0)	43 (87.8)	0.076
Present	6 (7.3)	0 (0.0)	6 (12.2)	
Technique complications (%)				
None	76 (92.7)	33 (100.0)	43 (87.8)	0.24
Bleeding	1 (1.2)	0 (0.0)	1 (2.0)	
Wire rupture	4 (4.9)	0 (0.0)	4 (8.2)	
Unreleased	1 (1.2)	0 (0.0)	1 (2.0)	
Occurrence of postoperative complications (%)				
Absent	67 (81.7)	27 (81.8)	40 (81.6)	1.000
Present	15 (18.3)	6 (18.2)	9 (18.4)	
Postoperative complications (%)				
None	67 (81.7)	27 (81.8)	40 (81.6)	0.452
Infection	2 (2.4)	0 (0.0)	2 (4.1)	
Migration	7 (8.5)	3 (9.1)	4 (8.2)	
Dislocation	4 (4.9)	3 (9.1)	1 (2.0)	
Connection trouble with the tube	2 (2.4)	0 (0.0)	2 (4.1)	

Abbreviation: SD, standard deviation.

#### 3.3.2 Postoperative Complications (Within 180 days)

Postoperative complications occurred in 18.2% (6/33) of Group N and 18.4% (9/49) of Group Z, with no significant difference observed between the groups (risk difference [Group Z − Group N]: 0.2%, 95% CI: −20.2–18.7, *p* = 1.00).

Major postoperative complications included stoma site infection (cellulitis) in two (4.1%) cases. Both cases occurred in Group Z. Subcutaneous or intraperitoneal migration (Group N, three cases [9.1%]; Group Z, four cases [8.2%]), and accidental removal (self‐removal or during care) (Group N, three cases [9.1%]; Group Z, one case [2.0%]), with no significant differences in incidence between the groups.

Both cases of postoperative stoma site infection occurred in Group Z, whereas no infections occurred in Group N. All infections were mild cellulitis treated with oral antibiotics without hospitalization.

### 3.4 Family Satisfaction

Family satisfaction scores did not differ significantly between groups (Group N: 3.88 ± 1.52 vs. Group Z: 3.94 ± 1.39; mean difference [Group Z − Group N]: 0.06, 95% CI: −0.71–0.59, *p* = 0.854).

### 3.5 Additional Considerations

Exchange methods (endoscopic vs. PEG scope) and ZERO device refusal rates were investigated, but no significant differences were observed between the groups.

## 4 Discussion

To our knowledge, this is the first retrospective observational analysis comparing the clinical outcomes of the newly developed Ideal Button ZERO with conventional Ideal Buttons. The key findings included a non‐significant trend toward higher procedure‐related complications and a significant reduction in procedure time in the ZERO group. We summarize the differences between the two devices (Table 5). Although the mean reduction in procedure time was modest (∼2 min), this difference may still reflect improved device operability and reduced patient discomfort.

**TABLE 5:** Comparison between the conventional IDEAL button and the IDEAL button ZERO.

Feature	Conventional IDEAL button	IDEAL button ZERO
Insertion mechanism	Direct bumper insertion	Capsule‐based enclosed insertion
Wire system	External wire removal	Internal wire encapsulation
Fistula tract resistance	Moderate	Reduced by capsule compression
Pain/discomfort during removal	Moderate–severe	Reduced
Risk of wire breakage	None	Initially higher
Button design	Flat, simple	Thicker
Intended benefit	Conventional exchange	Simplified, safer exchange with reduced resistance

### 4.1 Procedure‐related Complications

**FIGURE 3:**
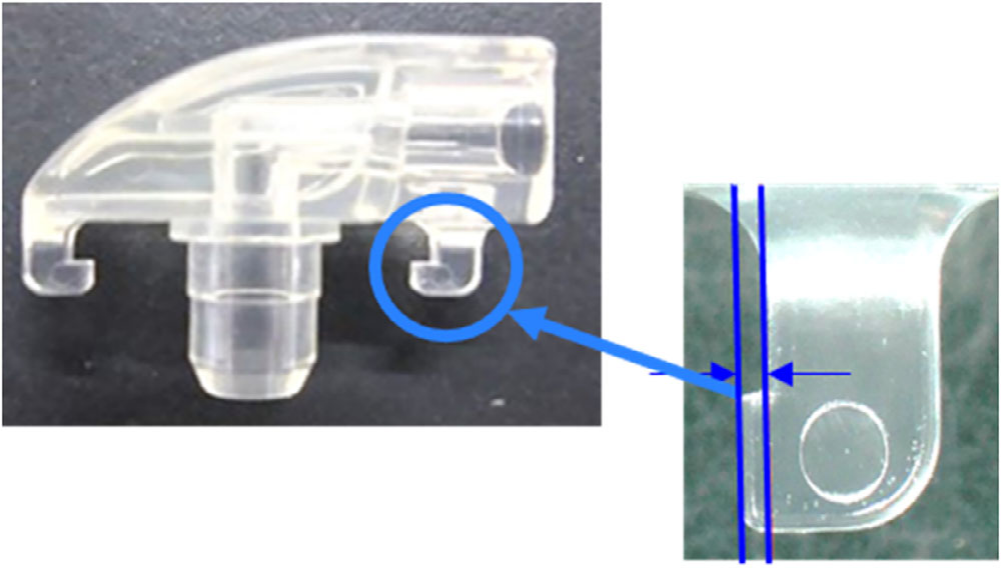
Nutrition tube connection design (the blue circle indicates the 0.3‐mm rear claw).

There is no evidence that morbidity directly related to the gastrostomy procedure is worse in patients with dementia, making patient factors an unlikely explanation for the observed complication rates. Several other factors may account for the higher procedure‐related complication rate in the ZERO group:

*Learning Curve Effect*: Learning curves are well recognized in novel medical device interventions [4, 11], with higher complication rates often observed during early adoption. As cases in the ZERO group began immediately after the June 2023 launch, limited healthcare provider proficiency may have contributed to the outcomes. Unlike conventional devices, the capsule mechanism of the ZERO requires different procedural steps, which may explain the higher incidence of intraoperative complications.
*Mechanical Device Characteristics*: Wire breakage, the most frequent complication (four cases), likely resulted from excessive pulling force. The manufacturer has since modified the device, shortening the rear claw length from 1 to 0.3 mm and strengthening the anchor mechanism to reduce wire dislodgment (Figure 3). These modifications are expected to improve outcomes. No differences were observed between the early and late study periods, and improved ZERO devices were not included in this study.
*Procedure Time Reduction*: Procedure times in Group Z were shorter, suggesting a potential advantage of the new device. The capsule mechanism may simplify previously complex endoscopic manipulations, reducing procedural time. Improved proficiency likely contributes to enhanced efficiency, and further reductions in procedure time are anticipated with increased experience. Shorter procedure times may reduce patient burden, improve healthcare worker efficiency, and lower medical costs.
*Postoperative Complications and Family Satisfaction*: Postoperative complication rates and family satisfaction scores showed no significant differences between the groups, suggesting that the improvements of the new device primarily affect intraoperative operability rather than long‐term outcomes.
  All postoperative infections occurred in Group Z. This may be partially explained by early operator unfamiliarity with the new device and the slightly thicker button design, which might have caused transient local pressure or minor leakage at the stoma site.
*Practical Considerations*: Despite its advantages, the practical drawbacks of the ZERO were noted. The device is thicker and larger than conventional PEG buttons, sometimes causing issues, such as catching on clothing or during patient mobility. Additionally, the nutrition connection tube requires a 180° rotation for connection, making the operation difficult for patients and older caregivers. Seven patients in this study switched from ZERO to conventional devices due to these difficulties.

Overall, these results provide important insights into the clinical implementation of the Ideal Button ZERO. While clear superiority over conventional devices has not been demonstrated, trends toward reduced procedure time and potential outcome improvements with increased proficiency suggest that this device may become valuable with appropriate educational and training systems. Comprehensive training programs, staged case implementation based on proficiency, and ongoing product improvement in collaboration with manufacturers are particularly recommended.

### 4.2 Study Limitations and Future Directions

This study has certain limitations. First, its single‐center, retrospective design introduces the potential for selection and information biases. Second, the relatively small sample size limits statistical power. Third, analyzing the learning curve for the new device was challenging.

Future directions include multicenter prospective studies with larger cohorts, stratified analyses based on provider proficiency, long‐term outcome evaluations, and prospective assessments incorporating patient‐reported pain outcomes.

In conclusion, the new Ideal Button ZERO demonstrated a significant reduction in procedure time compared with the conventional device; however, it showed a non‐significant trend toward higher procedure‐related complications. These findings suggest that operator proficiency and structured training programs will be crucial to realizing the potential benefits of the new device. Further multicenter prospective studies are warranted.

## Author Contributions

**Manuscript drafting**: Kazuya Miyaguchi. **Statistical analysis**: Maiko Osawa, Yuki Shiko, and Yohei Kawasaki. **Supervision**: Hisashi Matsumoto, Rie Shiomi, Yoshikazu Tsuzuki, Keiji Yamamoto, and Hiroyuki Imaeda. The final version of the manuscript was read and approved by all authors.

## Funding

The authors received specific funding for this work.

## Ethics Statement

**Approval of the Research Protocol by an Institutional Review Board**: The study protocol was in accordance with the tenets of the revised Declaration of Helsinki (1989) and was approved by the Saitama Medical University institutional review board (2025–047).

## Consent

Written informed consent was obtained from all patients.

## Conflicts of Interest

The authors declare no conflicts of interest.

## Data Availability

The data that support the findings of this study are available from the corresponding author upon reasonable request.
